# Diminished prospective mental representations of reward mediate reward learning strategies among youth with internalizing symptoms

**DOI:** 10.1017/S0033291723000478

**Published:** 2023-10

**Authors:** Josh M. Cisler, Amanda J. F. Tamman, Greg A. Fonzo

**Affiliations:** 1 Department of Psychiatry and Behavioral Sciences, Dell Medical School, University of Texas at Austin, USA; 2 Institute for Early Life Adversity Research, Dell Medical School, University of Texas at Austin, USA; 3 Menninger Department of Psychiatry and Behavioral Sciences, Baylor College of Medicine, Houston, TX, USA; 4 Center for Psychedelic Research and Therapy, Dell Medical School, University of Texas at Austin, USA

**Keywords:** Early life trauma, internalizing symptoms, MVPA, reinforcement learning, reward learning, striatum

## Abstract

**Background:**

Adolescent internalizing symptoms and trauma exposure have been linked with altered reward learning processes and decreased ventral striatal responses to rewarding cues. Recent computational work on decision-making highlights an important role for prospective representations of the imagined outcomes of different choices. This study tested whether internalizing symptoms and trauma exposure among youth impact the generation of prospective reward representations during decision-making and potentially mediate altered behavioral strategies during reward learning.

**Methods:**

Sixty-one adolescent females with varying exposure to interpersonal violence exposure (*n* = 31 with histories of physical or sexual assault) and severity of internalizing symptoms completed a social reward learning task during fMRI. Multivariate pattern analyses (MVPA) were used to decode neural reward representations at the time of choice.

**Results:**

MVPA demonstrated that rewarding outcomes could accurately be decoded within several large-scale distributed networks (e.g. frontoparietal and striatum networks), that these reward representations were reactivated prospectively at the time of choice in proportion to the expected probability of receiving reward, and that youth with behavioral strategies that favored exploiting high reward options demonstrated greater prospective generation of reward representations. Youth internalizing symptoms, but not trauma exposure characteristics, were negatively associated with both the behavioral strategy of exploiting high reward options as well as the prospective generation of reward representations in the striatum.

**Conclusions:**

These data suggest diminished prospective mental simulation of reward as a mechanism of altered reward learning strategies among youth with internalizing symptoms.

## Introduction

Early life trauma, including emotional, physical, and sexual abuse, is a well-established risk factor for multiple forms of mental and physical well-being (Dube et al., 2001, 2003; Felitti et al., 1998). While impact of early life trauma on behavioral and neurophysiological systems related to stress and threat responding are primary mechanisms explaining conferred risk for psychopathology (Mcewen, 2004; McLaughlin, Sheridan, Humphreys, Belsky, & Ellis, 2021; Nemeroff, 2016), there has been growing recognition and interest in the role of systems related to decision-making and reward learning as additional and non-mutually exclusive pathways to psychopathology (Fonzo, 2018; Hanson, Williams, Bangasser, & Peña, 2021; McLaughlin, DeCross, Jovanovic, & Tottenham, 2019). Further elaboration of specific mechanistic pathways will hopefully continue to inform development of prevention and intervention modalities.

Several lines of research support a mechanistic pathway of altered reward learning and decision-making mediating the relationship between early life trauma and psychopathology, particularly internalizing symptoms. Youth exposed to early life trauma learn reward contingencies more slowly and have decreased activation of striatum and dorsal anterior cingulate during reward learning tasks (Cisler et al., 2019; Gerin et al., 2017; Hanson, Hariri, & Williamson, 2015; Harms, Shannon Bowen, Hanson, & Pollak, 2018; Lenow, Scott Steele, Smitherman, Kilts, & Cisler, 2014). Similarly, youth with internalizing disorders demonstrate decreased striatal responses during the receipt and anticipation of reward (Auerbach, Admon, & Pizzagalli, 2014; Keren et al., 2018; Rappaport, Kandala, Luby, & Barch, 2020), consistent with altered neural reward responsiveness as a mechanism of observed clinical symptoms (e.g. anhedonia, avoidance of potentially rewarding activities, etc). Indeed, prospective studies demonstrate that decreased striatal reactivity to rewards predict development of future internalizing symptoms among youth (Hanson et al., 2015; Stringaris et al., 2015). While decreased striatal responses to reward are more consistently observed among depressed youth (Tang et al., 2022), reduced striatal activation to reward has also been observed in large samples of youth with anxiety disorders (Auerbach et al., 2022), and altered striatal response to reward also predicts anxiety symptom reduction during treatment among youth with anxiety disorders (Sequeira et al., 2021), possibly by enabling greater engagement with therapy.

The role of prospective episodic memory and mental simulation represent an emerging area of interest in the study of reward learning and decision-making (Biderman, Bakkour, & Shohamy, 2020; Dasgupta & Gershman, 2021; Mattar & Lengyel, 2022; Schacter, Benoit, & Szpunar, 2017; Sosa & Giocomo, 2021), though these processes have never been examined among at-risk youth. Numerous lines of research using animal and human models demonstrates that neural patterns associated with memory representations for the possible outcomes of a choice are activated at the time of choice as a form of mental simulation of future events (i.e. neural ‘preplay’) (Biderman et al., 2020; Doll, Duncan, Simon, Shohamy, & Daw, 2015; Schacter et al., 2017; Shadlen & Shohamy, 2016; Sosa & Giocomo, 2021; Widloski & Foster, 2022; Wikenheiser & Redish, 2015; Yu & Frank, 2015; Zielinski, Tang, & Jadhav, 2020). For example, memory representations for an aversive outcome become active prior to selecting amongst choices where an aversive outcome is possible and the magnitude of these representations predicts subsequent choices to avoid the expected aversive outcome (Castegnetti et al., 2020; Moughrabi et al., 2022). One emerging model explaining these phenomena posits that reactivation of memory representations reflects a prospective planning process, whereby the learner imagines possible outcomes for different branches of a decision tree and uses these imagined outcomes to inform selection of an appropriate response given the current context and goals (Biderman et al., 2020; Doll et al., 2015; Schacter et al., 2017). Further, experimental studies suggest that engaging imagined future rewarding outcomes increases reward-related neural activity in the medial prefrontal cortex (Peters & Büchel, 2010). Note that mental simulation of imagined outcomes as a mechanism of reward decision-making is a separate, though likely related process, to reward anticipation.

Testing the hypothesis of altered reactivation of reward representations at the time of choice among at risk youth has the potential to extend and complement prior work suggesting altered striatal and salience network activity during the anticipation and receipt of reward outcomes (Auerbach et al., 2022; Birn, Roeber, & Pollak, 2017; Cisler et al., 2019; Harms et al., 2018; Lenow et al., 2014). Indeed, understanding processes at the time of choice during laboratory tasks may help explain clinical behavior in this population, such as choices to behaviorally withdraw and/or avoid activities. For example, decreased mental simulation of reward might help explain behavioral withdrawal, such that youth who cannot engage a mental simulation of a rewarding outcome see little reason to exert effort to engage in the behavior. In the context of laboratory reinforcement learning tasks (e.g. bandit tasks), response selection is a separate, though related, process from response valuation. One concept related to selecting responses with varying degrees of expected value is the exploration-exploitation tradeoff (Daw, O'Doherty, Dayan, Seymour, & Dolan, 2006; Schulz & Gershman, 2019; Wilson, Bonawitz, Costa, & Ebitz, 2021). Exploitation broadly refers to a strategy that favors selecting responses that have a high expectation of value; exploration broadly refers to a strategy favoring a wider sampling of available response. Exploration has been differentiated into random exploration and information-directed exploitation (Schulz & Gershman, 2019; Wilson et al., 2021). The latter refers to a strategy of sampling amongst available choices for the explicit purpose of gaining information about those choices. The former refers to an ostensibly stochastic process underlying response selection, such that choice is uncoupled from both the choice's expected outcome probability and the value of gaining information about the environment by selecting that choice. Whereas younger children tend to show random exploration, adolescents show increasingly structured information-directed exploration (Meder, Wu, Schulz, & Ruggeri, 2021; Somerville et al., 2017). In the context of prospective memory representations for reward and mental simulation as a mechanism for decision-making, it is plausible that individual differences in random exploration are explained by individual differences in mental simulation for reward. For example, youth exposed to trauma and/or with internalizing symptoms with limited access to reward memory exemplars might be expected to make ostensibly stochastic decisions for reasons other than expected value due to their difficulty generating prospective reward representations.

No prior research has tested this hypothesis about prospective memory representations, with only limited and inconsistent prior computationally-driven behavioral investigations of choice strategies during reward learning among youth with trauma exposure and/or internalizing symptoms (Cisler et al., 2019; Harms et al., 2018; Humphreys et al., 2015; Sheridan et al., 2018). Some studies using foraging tasks suggest increased exploitation among adults with significant histories of early life adversity (Lenow, Constantino, Daw, & Phelps, 2017; Lloyd, McKay, & Furl, 2022). A large sample of previously institutionalized youth demonstrated greater exploitation compared to typically developing youth on a risky decision-making task (Humphreys et al., 2015), though this task may better reflect risk-taking (Humphreys, Lee, & Tottenham, 2013; Lejuez et al., 2002) than exploration. By contrast, one small prior study using a three-arm bandit task found increased choice stochasticity during social decision-making among assaulted adolescent girls (Lenow, Cisler, & Bush, 2015), and a larger study of youth with mixed histories of assault and clinical symptoms completing a similar task did not identify significant relationships between trauma exposure variables and exploration / exploitation strategies (Cisler et al., 2019). Among adults, a meta-analysis identified decreased reward sensitivity among depressed individuals (Huys, Pizzagalli, Bogdan, & Dayan, 2013), though as the authors note, their reward sensitivity parameter was mathematically interchangeable with an exploitation parameter, consistent with other research among depressed adults (Blanco, Otto, Maddox, Beevers, & Love, 2013; Dubois & Hauser, 2022). Accordingly, further investigation into choice selection strategies and their neurocircuitry mechanisms among youth exposed to trauma and/or with internalizing symptoms is necessary.

Here, we aim to investigate aberrant generation of prospective memory representations for reward and their relationships with reward learning strategies as well as trauma exposure and internalizing symptoms among youth.

## Methods

61 adolescent girls, age 11–17, participated in the study at two different sites: Little Rock, AR and the surrounding area (*n* = 26 participants; *n* = 13 exposed to assault), and Madison, WI and the surrounding area (*n* = 35 participants; *n* = 18 exposed to assault). Participants were recruited from community-wide advertising, social medial posting, and outpatient mental health clinic referrals. Healthy controls were recruited based on absence of current mental health disorders, trauma exposure, and psychiatric treatment histories. Inclusion criteria for the assaulted group consisted of a history of directly experienced physical or sexual assault that the participant could remember. Exclusion criteria for all participants included histories of psychotic symptoms, developmental disorders, major medical disorders, MRI contraindications, pregnancy, history of loss of consciousness greater than 10 min. Psychotropic medication was not exclusionary for the assaulted adolescents; however, a stable dose on any medication for at least 4 weeks was required. Table 1 presents clinical and demographic characteristics. Imaging data were excluded for one participant, an assaulted girl, due to excessive head motion, and imaging data were unusable from two participants, both controls, due to technical error during scanning. The imaging analyses included 58 participants and all participants' data were used in behavioral analyses. All study procedures were approved by the local IRB committees.

**Table 1.** Clinical and demographic characteristics of the participants

Variable	Control *N* = 30	Assault *N* = 31	*p* values
Age (yr)	14.9 (2.3)	15.7 (1.5)	0.083
Verbal IQ	112.7 (29.5)	101.2 (17.9)	0.070
Ethnicity (%)			0.431
White	60.0	54.8	
Black	20.0	19.4	
Asian	3.3	0.0	
Hispanic, Latina	3.3	16.1	
Other	13.3	9.7	
Age at first assault (yr)	–	8.8 (4.8)	–
Age at last assault (yr)	–	14.2 (3.5)	–
Time since last assault (yr)	–	1.5 (2.5)	–
Direct assault types (#)	–	3.5 (2.1)	–
Assault Exposure (%)			
Sexual assault	–	80.6	–
Physical assault	–	74.2	–
CAPS Total	–	44.4 (30.4)	–
CBCL Anxiety	2.38 (2.39)	7.76 (6.23)	**<0.001**
CBCL Depression	1.38 (1.91)	4.62 (3.3)	**0.002**
CBCL Somatic Complaints	1.33 (1.83)	4 (3.46)	**<0.001**
CBCL Internalizing	5.08 (4.94)	16.38 (11.19)	**<0.001**
CTQ Total	29.4 (5.0)	50.8 (17.0)	**<0.001**
Current Psychopathology (%)			
PTSD	–	53.3	–
MDD	–	25.8	–
Dysthymia	–	35.5	–
Bipolar I or II	–	3.2	–
Agoraphobia	–	54.8	–
Social phobia	–	22.6	–
SUD	–	19.4	–
GAD	–	29.0	–
Panic disorder	–	16.1	–
Separation anxiety	–	9.7	–
Specific phobia	–	19.4	–
OCD	–	22.6	–
Psychiatric Medications (%)			
Antidepressants	–	6.5	–
SSRI	–	38.7	–
SNRI	–	3.2	–
NDRI	–	3.2	–
Benzodiazepine	–	3.2	–
Mood stabilizer	–	12.9	–
Anticonvulsant	–	3.2	–
Stimulants	–	6.5	–
Any psychiatric medication	–	58.1	–

Note. IQ was assessed from the Receptive One-Word Picture Vocabulary Test. CTQ, Childhood Trauma Questionnaire; UCLA PTSD RI, UCLA PTSD Reaction Index; CAPS, Clinician Administered PTSD Scale; CBCL, Child Behavior Checklist; CBCL values represent raw values; DERS, Difficulties in Emotion Regulation Scale. Psychopathology was assessed using the Mini-International Neuropsychiatric Interview for Children and Adolescents (MINI Kid). Bolded values represent a statistical difference, two-tailed (*p* < 0.05).

Portions of these data pertaining to the impact of trauma characteristics on outcome processing (i.e. prediction error encoding and latent state belief updating) have previously been published (Cisler et al., 2019; Letkiewicz, Cochran, Privratsky, James, & Cisler, 2022). The present analysis is a novel investigation of multivariate representations at the time of choice as a function of trauma exposure characteristics and internalizing symptoms.

### Assessments

Internalizing symptoms were assessed with the caregiver-rated Child Behavior Checklist (Achenbach, 1991) (CBCL), consisting of the sum of anxiety, depression, and somatic concern subscales. The Clinician Administered PTSD Scale, Child and Adolescent Version (CAPS) (Nader, Blake, Pynoos, Newman, & Weathers, n.d.), was used to assess PTSD symptoms, and PTSD diagnoses followed definitions established by prior studies among youth (Cohen, Deblinger, Mannarino, & Steer, 2004). The Mini-International Neuropsychiatric Interview for Children and Adolescents (MINI-KID) (Sheehan et al., 2010) assessed for current and lifetime comorbid mental health disorders. Assault exposure histories were defined using the trauma assessment section of the National Survey of Adolescents (NSA) (Kilpatrick et al., 2003). Participants also completed the Childhood Trauma Questionnaire (Bernstein et al., 1994), providing a continuous measure of the total severity of early life maltreatment and trauma across the domains of emotional abuse, physical abuse, sexual abuse, emotional neglect, and physical neglect. We also assessed participants' verbal IQ (Brownell, 2000).

### MRI acquisition and image preprocessing

See online Supplemental material.

#### Reinforcement learning task

**Fig. 1.**
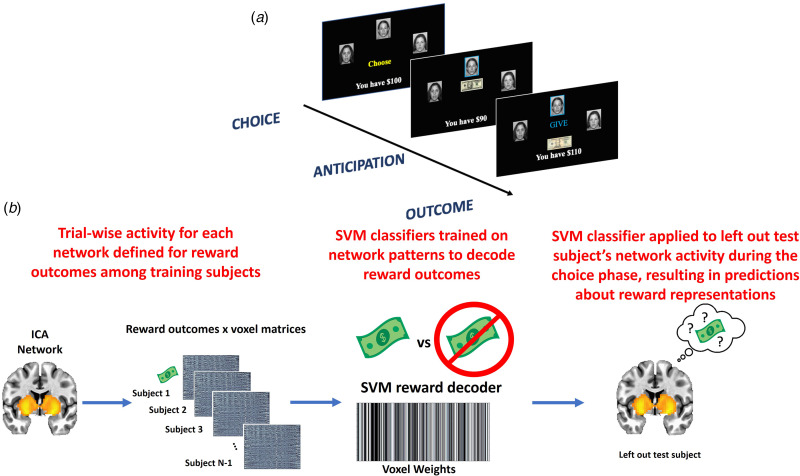
(a) Depiction of the social reward three-arm bandit task. Participants completed 90 trials. Trials began with presentation of three faces and participants chose one face in which to invest $10. The choice phase lasted until participants made a selection, which was then indicated with a blue box around it for 1s. An anticipation phase followed while they waited for the outcome of the choice, which consisted of a jittered fixation cross for 1.5–3s. The outcome phase was subsequently displayed and consisted of binary return of either $20 (net increase of $10) or no return (net loss of $10). The outcome phase presented the outcome of the trial (win or loss) for 2s, updated the points total for 1s, followed by a jittered fixation cross of 1.5–3s prior to starting the next trial. (b) Depiction of the MVPA pipeline. For each ICA network separately, trial × voxel matrices of beta coefficients are created for all participants except one left out participant separately for reward outcomes during the task. Support vector machine classifiers are then trained on these data, resulting in a decoder for reward outcomes. Next, this reward decoder is applied to the trial × voxel matrix of beta coefficients at the time choice for the participant that was left out of the training. This results in a prediction about the degree to which the reward representations are active at the time of choice, which can be compared to the magnitude of reward the participant was expecting for that given choice. This process is repeated until each participant has served as the left-out test participant.

Participants completed a three-arm bandit task using social stimuli (Fig. 1a) in a counterbalanced order. Participants were directed to give $10 to one of three mock people who returned either $20 or $0. The probabilities of positive returns varied by arm, either 80, 50, or 20%. Probabilities changed across the mock people every 30 trials, for a total of 90 trials. The same faces were used for all trials. Participants were informed that their compensation would be proportional to task performance. Additional information is provided in online Supplemental material and Fig. 1 legend.

*Modeling Reinforcement Learning.* Behavior during the RL tasks was modeled using versions of the Rescorla-Wagner (RW) model (Sutton & Barto, 1998). Consistent with prior research (Hauser, Iannaccone, Walitza, Brandeis, & Brem, 2015; Ross, Lenow, Kilts, & Cisler, 2018), four different RW-based models were tested, which manipulated whether the model updated the expected value of the unchosen option (Hauser et al., 2015) and whether the model was risk-sensitive (Niv, Edlund, Dayan, & O'Doherty, 2012). Expected reward values for each arm were transformed into choice probabilities using a softmax function, providing individually varying *β*s that reflect the degree to which an individual's choices are driven by reward expectations. Model fitting was conducted using hierarchical Bayesian inference (Piray, Dezfouli, Heskes, Frank, & Daw, 2019). See online Supplemental material for additional information.

*Independent Component Analysis.* An Independent Component Analysis (Calhoun, Adali, Pearlson, & Pekar, 2001) (ICA) with a model order of 35 components was conducted on the full voxelwise fMRI timecourses. This model order delivered a good balance between component reliability estimated across 50 ICASSO iterations and interpretability of canonical networks. 8 of the 35 components were deemed functional networks of interest after visual inspection (see Fig. 3a below). Components arising from artifacts of head motion or CSF and components of non-interest (i.e. motor, sensorimotor, and visual networks), which are not hypothesized to be relevant for understanding trauma, internalizing symptoms, reward learning, or PTSD (Auerbach et al., 2022; Patel, Spreng, Shin, & Girard, 2012), were excluded.

#### Multivariate pattern analyses of prospective mental representations during choice

Figure 1b provides an overview of the analytical approach, which is in direct accord with our previous MVPA investigation of prospective representations of reward and threat as a mechanism of decision-making (Moughrabi et al., 2022). The first step was to demonstrate that network activity patterns at the time of reward delivery could accurately be decoded. Each participants' trial-by-trial activation patterns at the time of reward delivery were characterized using 3 dLSS. The timepoint × voxel matrices were centered within each timepoint to ensure no differences in overall activation across trials. Support vector machines (SVM), using a radial basis function kernel implemented in Matlab through libsvm (Chang & Lin, 2011), were used to decode reward outcomes (binary classification). We established the accuracy of the decoders using leave-one-out cross-validation across subjects (i.e. one subject was designated as the left-out test subject, decoders were trained on the remaining test subjects (i.e. N-1 sample size), then the decoder was tested on the independent left-out subject's data. This process was repeated until all subjects served as the left-out test subject. The reward decoder accuracy was defined as the mean of sensitivity and specificity.

After testing accuracy of the reward decoders, the next step was to apply the reward decoders to participant's data at the time of choice. 3dLSS was used to define trial-by-trial activation at the time of choice. A leave-one-out approach was used, such that a subject was designated as the left-out test subject, the reward decoders were trained on all remaining participants' reward outcome data, and the resulting reward decoders were applied to the left-out participant's choice data. This process was repeated for each subject. This resulted in hyperplane distances representing the degree to which the trained multivariate patterns (reward outcomes) were active at the time of choice. This process was repeated separately for each ICA network of interest, resulting in unique predictions (i.e. hyperplane distances) about reward representation activation for each separate network.

Our primary interest was investigating coupling between prospective reward representation at the time of choice and the expected reward value, derived from the computational model, of the chosen arm. That is, the degree to which a youth is expecting reward for a given choice should be related to the degree of activation of prospective reward representations at the time of that choice. To test this hypothesis, we conducted linear mixed effects models (LMEMs), in which trial-by-trial reward expectations (*V* of the chosen arm from the fitted computational model) were regressed onto the trial-by-trial hyperplane distances. We stringently controlled for multiple comparisons across the 8 ICA networks with Bonferroni correction, resulting in a corrected alpha of *p* = 0.0063. These models included covariates for age, IQ, and head motion. We included an additional covariate for each subject's cross-validation reward decoding accuracy (Greene et al., 2022). Main results without these covariates, which remain essentially unchanged, are included in the online Supplemental material. We modeled subject and site as random effects in all models, with subject nested within site.

LMEMs then tested whether individual differences moderated the coupling between prospective reward representations (hyperplane distances) and expected reward, using identical models and including interaction terms with the individual difference variable. We first investigated associations with trauma exposure (continuous measure of log transformed CTQ total score or dichotomous assault exposure in separate LMEMs) on coupling of reward representations with expected reward. Subsequent models then retained trauma exposure severity (log transformed CTQ total score) as a covariate and tested CBCL internalizing symptoms, PTSD symptoms, and decomposed CBCL internalizing symptoms into its constituent scales of depression, anxiety, and somatic complaints. While the study recruited controls and assaulted participants as separate groups, given the continuous distributions of CTQ total scores and internalizing symptoms (online Supplemental Fig. S1), we opted to use these continuous variables among the entire sample to conserve statistical power. Bonferroni correction again controlled for family-wise multiple comparisons. Mediation analyses tested the significance of hypothesized indirect effects through bootstrapping with replacement using 50 000 iterations following contemporary recommendations for mediation analyses (Hayes & Rockwood, 2017).

## Results

### Relationship between learning parameters and clinical characteristics

**Fig. 2.**
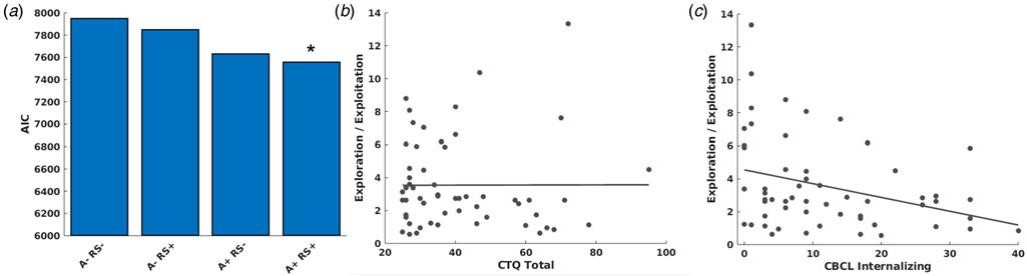
(a) Akaike Information Criterion values of model fit for the compared models. We tested a factorial manipulation of anticorrelated or not anticorrelated models (denoted with A+ or A−) and risk sensitive or not risk sensitivity models (denoted with RS+ or RS−). Consistent with our past studies using Matlab's fmincon for model fitting (Cisler et al., 2019; Ross et al., 2018), our updated approach using hierarchical Bayesian inference (Piray et al., 2019) similarly demonstrated the anticorrelated and risk sensitive model fit the data best. (b) There were no relationships between Childhood Trauma Questionnaire total severity scores and softmax *β*s, representing individual differences in exploitation / exploration strategies on the task. (c) There was a significant inverse relationship between CBCL internalizing symptoms and softmax *β*s, suggesting decreased exploitative behavior among those with greater internalizing symptoms.

We first investigated relationships between clinical variables and softmax *β*s from the best fitting model (Fig. 2a). Regression models, conducted separately for CTQ total scores and dichotomous control *v.* assault group comparisons, did not demonstrate significant relationships between softmax *β*s and CTQ total scores, *p* = 0.76 (Fig. 2b) nor dichotomous control *v.* assaulted group comparisons, *p* = 0.58. When controlling for CTQ total scores, identical models demonstrated that CBCL internalizing symptoms were significantly related to softmax *β*s, *t*(51) = −3.15, *p* = 0.003 (Fig. 2c), demonstrating decreased choice preference for high reward options and greater response stochasticity. Decomposing internalizing symptoms in separate models demonstrated similar relationships with depression symptoms, *t*(51) = −2.70, *p* = 0.009, anxiety, *t*(51) = −3.2, *p* = 0.002, and somatic complaints, *t*(51) = −2.37, *p* = 0.02 (online Supplemental Figs S1a–c). CAPS total symptom severity scores among the traumatized youth were similarly negatively related to softmax *β*s, *t*(25) = −2.54, *p* = 0.018. There were no relationships between trauma characteristics and clinical variables with positive or negative learning rates (*p*s > 0.3).

### Multivariate representations for reward at the time of choice and coupling with reward expectations

**Fig. 3.**
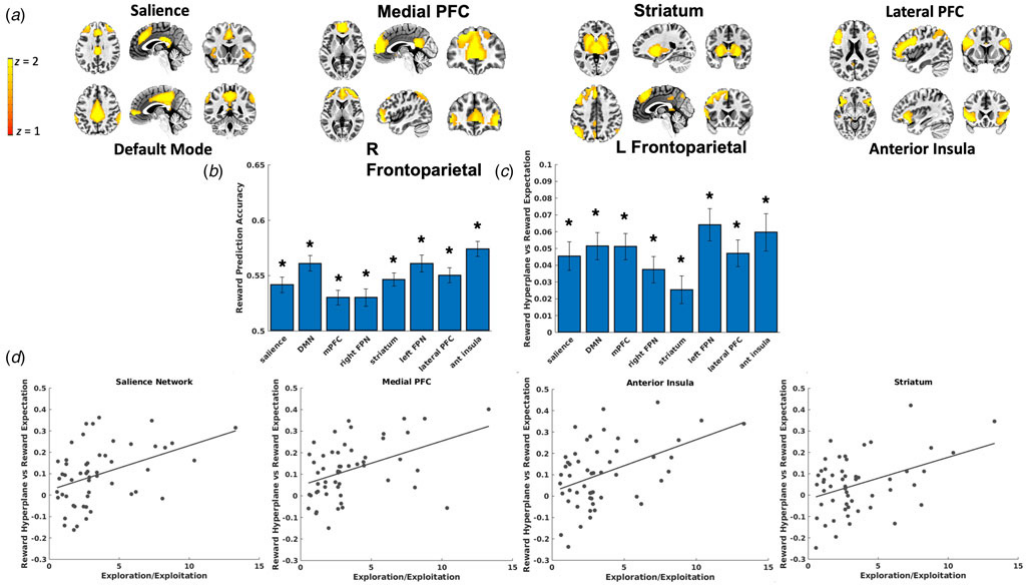
(a) Depiction of spatial maps from the Independent Component Analysis. (b) Reward decoding performance for each ICA network. Decoding performance was defined as the mean of sensitivity and specificity in correctly classifying reward outcomes from the left-out participant using the model trained on the remaining participants' data. (c) *β* coefficients reflecting the degree to which value expectation, derived from the computational model, of the chosen arm on the task predicted the magnitude of MVPA predicted reward presentations (i.e. SVM hyperplane predictions) at the time of choice. All networks demonstrated significant coupling between reward expectation and magnitude of reward representations when controlling for multiple comparisons. (d). ICA networks demonstrating significant interactions between softmax *β*s and coupling between reward expectation and magnitude of reward representations (i.e. SVM hyperplane predictions), suggesting that those who generated greater reward representations in proportion to expected reward also tended to use behavioral strategies to exploit high reward arms.

Leave-one-out cross-validation accuracy for reward outcomes was above chance for all ICA networks (Fig. 3b), demonstrating that reward (*v.* loss) outcomes in a left-out participant could accurately be decoded from the other participants' patterns of voxel activity. We also observed that classifier cross-validation accuracy was not correlated with trauma characteristics (*p*s > 0.31 for assault group, *p*s > 0.47 for CTQ total score), internalizing symptoms (*p*s > 0.19), or PTSD symptom severity (*p*s > 0.6), suggesting that decoded reward representations were equally accurate regardless of trauma or clinical symptoms.

SVM classifiers were then applied to left-out participants' voxel patterns at the time of choice, resulting in trial-by-trial predictions about the degree to which reward representations were active while the participant contemplated which arm of the task to select. LMEMs tested the degree to which these trial-by-trial prospective reward representations were coupled with trial-by-trial reward expectations (i.e. *V*) derived from the computational model fit to participants' observed behavior. These models demonstrated that prospective reward representations in each of the tested networks were strongly coupled with expected reward for the chosen arm (Fig. 3c).

We next tested whether this coupling between prospective reward representations and expected reward varied as a function of behavioral strategies on the task. LMEMs demonstrated that coupling between reward representations and expected reward was positively associated with softmax *β*s in the salience, *t*(4690) = 3.22, *p* = 0.001, medial PFC, *t*(4690) = 3.88, *p* < 0.001, anterior insula, *t*(4690) = 3.41, *p* < 0.001, and striatum networks, *t*(4690) = 3.39, *p* < 0.001 (Fig. 3d), such that individuals who generated greater prospective reward representations in proportion to the expected reward probabilities of the chosen arm also demonstrated behavioral strategies favoring the selection of high value arms.

#### Associations among clinical characteristics and coupling between reward representations and expected reward

**Fig. 4.**
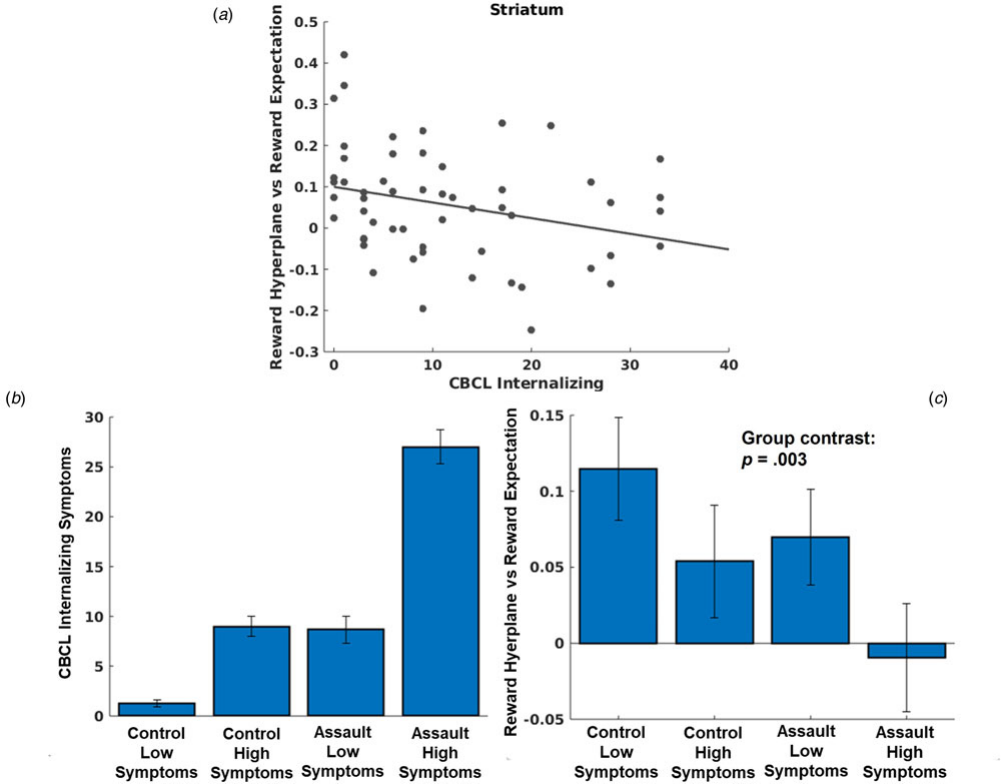
(a) Scatter plot depicting relationship between CBCL internalizing symptoms and coupling between MVPA reward representations during choice and reward expectations. (b) Even though we controlled for CTQ total severity in our primary analyses, we conducted an additional analysis differentiating effects of assault exposure and internalizing symptoms. We used a median split to identify control adolescents with low v. high internalizing symptoms, and separately used a median split to identify assaulted adolescents with low v. high internalizing symptoms. Separating the sample in this manner allows differentiation of impacts due to assault exposure and internalizing symptoms. If coupling of prospective reward representations in the striatum were more strongly associated with assault exposure, we would expect that both assault groups would demonstrate impairment relative to both control groups, with relative homogeneity within groups. By contrast, if coupling of prospective reward representations in the striatum were more strongly associated with internalizing symptoms, we would instead expect coupling of prospective reward representations to follow the pattern of internalizing symptoms across the groups in accordance with panel B. (c) As can be seen in Fig. 4c, individual differences in coupling with prospective reward representations clearly tracked individual differences in internalizing symptoms and not assault exposure, *t*(51) = −3.14, *p* = 0.003 (regression model with group coded as follows in accordance with differences in CBCL internalizing symptoms [see panel B]: control low symptoms = 0, control high symptoms = 1; assault low symptoms = 1, assault high symptoms = 2).

LMEMs demonstrated that greater CBCL internalizing symptoms was associated with de-coupling of reward expectations for a chosen arm and activation of prospective reward representations in the striatum network, *t*(4847) = −3.66, *p* < 0.001 (Fig. 4a). Additional models decomposing CBCL internalizing symptoms demonstrated similar relationships with depression, *t*(4847) = 3.94, *p* = 0.001, anxiety, *t*(4847) = 3.07, *p* = 0.002, and somatic complaints, *t*(4847) = −2.01, *p* = 0.04. Neither trauma characteristics (all *p* > 0.42 for CTQ total score; all *p* > 0.06 for assault group comparisons) nor PTSD symptom severity among the assaulted adolescents (all *p* > 0.048) were associated with coupling of prospective reward representations and reward expectations in any network when controlling for multiple comparisons. While these models controlled for overall trauma severity (CTQ total score), we conducted an additional post-hoc analysis to differentiate associations with assault exposure (i.e. the variable used for inclusion into the study) and internalizing symptoms (see Fig. 4b and 4c).

As an additional test of specificity, we demonstrated that internalizing symptoms, but not externalizing symptoms, were related to altered coupling of reward representations in the striatum (see online Supplemental material).

#### Prospective reward representations mediate the association between internalizing symptoms and behavioral strategies during learning

**Fig. 5.**
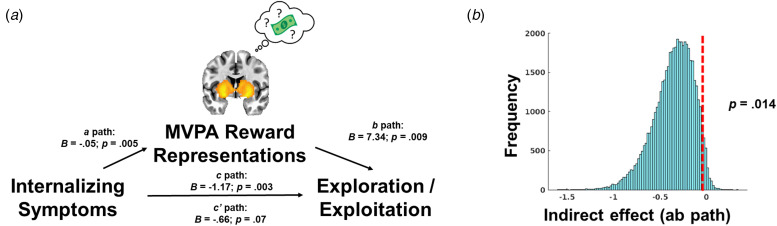
(a) Graphical depiction of mediation model, where internalizing symptoms predict decreased coupling between MVPA reward representations and expectations of reward in the striatum (i.e. path a), and decreased coupling of reward representations in the striatum predict decreased choices to exploit high reward arms on the task (i.e. path b). Path c refers to the total effect of internalizing symptoms on behavioral strategies on the task, and path c’ refers to the direct effect after accounting for the indirect effect (i.e. path ab) through MVPA reward representations. (b). The significance of the indirect effect was tested through 50 000 bootstrap iterations and demonstrating that the 95% confidence interval does not include zero.

We statistically tested whether coupling between prospective reward representations and reward expectation in the striatum mediated the association internalizing symptoms and softmax *β*s (Fig. 5a). We observed a significant indirect effect of internalizing symptoms through prospective reward representations in the striatum when tested through bootstrapping with 50 000 iterations (*p* = 0.014, ab path B = −0.36, 95% CI −0.76 to −0.055 (Fig. 5b). Decomposing internalizing symptoms, the indirect effect mediating pathway was also significant for depression symptoms (*p* = 0.013, ab path B = −0.51, 95% CI −1.07 to −0.085), anxiety symptoms (*p* = 0.014, ab path B = −0.39, 95% CI −0.85 to −0.055), but not somatic complaints (*p* = 0.067, ab path B = −0.26, 95% CI −0.66 to 0.013) (online Supplemental Figs S1d–f).

#### Ruling out site differences as confound

While we explicitly modeled site as a random factor in all analyses, we conducted additional analyses stratifying by site. As indicated in online Supplemental Figs S2a–c, effects were comparable at both sites and interaction terms testing significant differences in effects between sites were all non-significant (*p* >0.19).

## Discussion

We observed that internalizing symptoms among youth, but not child maltreatment or assault exposure, were related to a particular behavioral strategy during the task. Whereas youth with lower internalizing symptoms favored selecting task arms with higher expected value, youth with higher internalizing symptoms had less preference for selecting arms with higher expected value and instead demonstrated greater stochasticity in their choices. While softmax *β*s are linked with the well-known exploration/exploitation tradeoff, recent work on choice models during decision-making differentiates between directed and random exploration (Schulz & Gershman, 2019; Wilson et al., 2021). The former is exploration to obtain valuable information, whereas the latter reflects random noise in the decision-making process and is more akin to behavior captured by lower softmax *β*s. As such, the behavioral strategy observed among youth with higher internalizing symptoms appears less driven by expected reward probabilities and instead reflects underlying stochasticity in response selection.

To probe the mechanisms of this decision-making process and its relationship to reward expectations, we tested whether prospective representations of reward at the time of choice were coupled with expectations of reward. Consistent with hypotheses and the growing literature demonstrating a role for prospective memory representations as a fundamental mechanism of decision-making (Biderman et al., 2020; Doll et al., 2015; Gillespie et al., 2021; Moughrabi et al., 2022; Schacter et al., 2017), we observed significant coupling between reward expectations and magnitude of prospective reward representations. Our observation that multiple networks demonstrated significant coupling highlights a distributed network for reward encoding and is analogous to recent observations of the distributed, rather than localized, networks that encode subjective fear (Zhou et al., 2021). Further, coupling in the salience, medial PFC, anterior insula, and striatum networks was strongly associated with behavioral strategies characterized by favoring the selection of arms with higher expected value. That is, youth who favored choosing high reward arms also generated greater prospective representations of reward towards high reward arms. Recent interest has increased in understanding mechanisms underlying noise in decision-making (Collins & Shenhav, 2022; Schulz & Gershman, 2019; Wilson et al., 2021), and the current data, though correlational, support prospective representations of reward as a mechanism supporting a behavioral strategy characterized by favoring choices with higher expected value.

Next, we demonstrated that internalizing symptoms, but not assault exposure or maltreatment characteristics, were associated with less coupling between reward expectations and prospective representations of reward in the striatum network. Further, a statistical mediation model supported decreased coupling between reward expectations and prospective representations of reward as a mechanism mediating the association between internalizing symptoms and softmax *β*s. In this hypothesized model, the probability of reward for a given action does not engage a prospective representation for reward in the striatum among youth with internalizing symptoms. Consequently, youth with internalizing symptoms make decisions that are less governed by the likelihood of reward. These altered mechanisms of decision-making may help explain real-world behavior among youth with internalizing symptoms. For example, youth with depression symptoms may be biased to behaviorally withdraw and avoid ostensibly rewarding activities (e.g. social activities, going to school, extracurricular activities) due to a lack of generation of prospective mental representations of possible rewarding/meaningful occurrences during those activities.

The observation that internalizing symptoms, but not early life trauma that is a robust risk factor for internalizing symptoms, was related to the brain and behavioral alterations suggests these novel deficits in prospection are more strongly linked with the expression of psychopathology rather than risk for psychopathology. While prior research and theory suggests a link between childhood trauma and altered reward learning (Blair et al., 2022; Hanson et al., 2015; McLaughlin & Sheridan, 2016), it is not readily discernable why this link was not detected in the current study. It could be that prospective representations in the striatum are uniquely related to internalizing symptoms, whereas outcome processing of rewards is more linked with early life trauma (Cisler et al., 2019; Letkiewicz et al., 2022). Future research with larger sample sizes is necessary to continue to differentiate the unique impacts of trauma *v.* psychopathology on the various facets of reward learning and decision-making.

To our knowledge, this is the first demonstration of prospective multivariate representations of reward in the striatum as a possible mechanism of altered decision-making among youth with internalizing symptoms. Nonetheless, these data are fully consistent with related prior work demonstrating altered striatal activation during the anticipation and receipt of reward among youth with internalizing symptoms (Auerbach et al., 2022; Stringaris et al., 2015), behavioral inhibition (Guyer et al., 2014), and adults with mood and anxiety disorders (Cooper, Arulpragasam, & Treadway, 2018) and provide further support for emerging models emphasizing the role of altered decision-making for reward as a mechanism of psychopathology following trauma (Cisler & Herringa, 2021; Fonzo, 2018; McLaughlin et al., 2019; McLaughlin, Colich, Rodman, & Weissman, 2020). While we observed associations between internalizing symptoms and prospective reward representations in the striatum, it will be important to investigate additional brain regions and networks associated with episodic future thinking and reward [e.g. medial PFC, hippocampus, etc., (Peters & Büchel, 2010; Schacter et al., 2017)] and link these mechanisms with treatment response (Berwian et al., 2020; Webb, Murray, Tierney, Forbes, & Pizzagalli, 2022).

The current study is not without limitation. The sample was limited to adolescent girls and generalization to males and adults needs to be established. We used a relatively simple three-arm bandit task of social reward learning with binary outcomes, and the degree to which the results generalize to more complex task [e.g. two stage Markov task (Daw, Gershman, Seymour, Dayan, & Dolan, 2011)] needs to be tested. Our sample was recruited based on the presence of assault exposure, and while this resulted in a natural variation in the degree of internalizing symptoms in the current sample, testing among explicitly defined groups of youth with anxiety and depressive disorders is needed. Further, the effects we observed were limited to caregiver-report and future studies should seek to expand effects to additional modes of assessment.

## Supplementary material

For supplementary material accompanying this paper visit https://doi.org/10.1017/S0033291723000478. click here to view supplementary material
