# Supplementary material for: Diminished prospective mental representations of reward mediate reward learning strategies among youth with internalizing symptoms
Source: Psychol Med. 2023 Mar 7;53(14):6910–20. doi: 10.1017/S0033291723000478 (PMC10600826; doi:10.1017/S0033291723000478)
Supplement: Supplementary file 1 [file S0033291723000478sup001.docx]

*Reinforcement Learning Tasks*

Participants completed a three-arm bandit task using social stimuli (Figure 1). During the task, participants were directed to select among three mock people in which to invest $10, and the mock person either returned $20 or $0. The probabilities of positive returns were either 80%, 50%, or 20%, and probabilities across the mock people changed every 30 trials, for a total of 90 trials. Participants were told their study compensation would be proportional to their performance on the task. During the decision-phase and until the participant made a button press, the task presented the faces and the participant’s current amount of winnings. The interval/anticipation phase involved the chosen option being indicated by a surrounding colored box for 1s, followed by a jittered fixation cross for 1.5-3s. Finally, the outcome phase presented the outcome of the trial for 2s, followed by a jittered fixation cross for 1.5-3s. No participants needed to be removed from analyses due to poor performance (i.e., less than chance performance).

*Modeling reinforcement learning*.

We tested different adaptations of the RW model. The base RW model updates expected value of a chosen option (i.e. reward expectation), *V,* based on the magnitude of the prediction error, δ (observed outcome – *V*), scaled by a learning rate α (ranging from 0-1): V_t+1_=V_t_+ δ * α. The learning rate, α, controls the speed of updating *V*, with higher learning rates resulting in faster changes in expected value. A softmax function transformed *V* into action probabilities by using an exploration / exploitation β parameter. Higher softmax β represent a tendency to exploit high value responses / respond more consistently; lower softmax β represent a tendency to explore lower value options / respond more inconsistently. The anticorrelated version of the model updates the unchosen arms in the opposite direction as the chosen arm (e.g., increasing expectation for the chosen arm results in reciprocal decreasing expectations in the unchosen arms). The risk sensitive models include separate learning rates for positive prediction errors (i.e., rewarded trials) and negative prediction errors (i.e., loss trials). Model fitting was conducted using Hierarchical Bayesian Inference as implemented within the CBM toolbox (Piray, Dezfouli, Heskes, Frank, & Daw, 2019).

*MRI acquisition and image preprocessing*

At the Arkansas site, fMRI data were acquired on a Philips Achieva 3T X-series scanner using a 32-channel headcoil. T1-weighted anatomic images were acquired with a MP-RAGE sequence (matrix = 192 × 192, 160 sagittal slices, TR/TE/FA = 7.5ms/3.7ms/9°, FOV = 256, 256, 160, final resolution = 1 × 1 × 1 mm resolution). Echo planar imaging (EPI) sequences were used to collect the functional images using the following sequence parameters: TR/TE/FA = 2000 ms/30 ms/90°, FOV = 240 × 240 mm, matrix = 80 × 80, 37 axial slices (parallel to AC–PC plane to minimize OFC signal artifact), slice thickness = 2.5 mm, and final resolution of 3 × 3 × 3 mm.

At the UW-Madison site, fMRI data were acquired on a GE MR750 3T scanner using an 8-channel headcoil. T1-weighted anatomic images were acquired with a MP-RAGE sequence (matrix = 256x256, 156 axial slices, TR/TE/FA = 8.2ms/3.2ms/12°, FOV = 25.6cm, final resolution = 1x1x1mm). EPI sequences used to collect the functional images used the following parameters: TR/TE/FA = 2000ms/ 25 ms/ 60^0^, FOV = 24 cm, matrix = 64 x 64, 40 sagittal slices, slice thickness = 4 mm, original resolution was 4 x 3.75 x 3.75 mm, and images were resampled to match the resolution of the UAMS data of 3x3x3mm.

Image preprocessing followed standard steps and was completed using AFNI software in the following order. Images underwent despiking, slice timing correction, deobliquing, motion correction using rigid body alignment, alignment to participant’s normalized anatomical images, spatial smoothing using an 8 mm FWHM Gaussian filter (AFNIs 3dBlurToFWHM that estimates the amount of smoothing to add to each dataset to result in the desired level of final smoothing), detrending, low frequency (128 s) bandpass filtering, and rescaling into percent signal change. Images were normalized using the MNI 152 template brain using non-linear methods (AFNIs 3dQwarp implemented within @SSwarper for the anatomical images and 3dNwarpApply for functional images). Following recommendations (Power et al., 2014; Siegel et al., 2014), we corrected for head motion-related signal artifacts by using motion regressors derived from Volterra expansion, consisting of [R R^2^ R_t-1_ R^2^_t-1_], where R refers to each of the 6 motion parameters, and separate regressors for mean signal in the CSF and WM. This step was implemented directly after motion correction and normalization of the EPI images in the image preprocessing stream. Additionally, we censored TRs from the first-level GLMs based on threshold of framewise displacement (FD) > 0.4. FD refers to the sum of the absolute value of temporal differences across the 6 motion parameters; thus, a cut-off of 0.4 results in censoring TRs where the participant moved, in total across the 6 parameters, more than ~0.4 mm plus the immediately following TR (to account for delayed effects of motion artifact). Additionally, we censored isolated TRs where the preceding and following TRs were censored, and we censored entire runs if 50% or more of TRs within that run were censored. This led to the removal of 1 participant. Groups did not differ in head motion (*p* > .23).

*Main analyses without covariates*

When not considering age or IQ, CBCL internalizing symptoms continued to strongly associated with Softmax βs: *t*(53)=-3.52, *p*<.001.

When not considering age, IQ, head motion, or cross-validation accuracy, MVPA reward representation coupling with reward expectations continued to significantly associate with Softmax βs in the salience network, *t*(4694)=3.22, *p*=.001, medial PFC, *t*(4694)=3.88, *p*<.001, anterior insula, *t*(4694)=3.41, *p*<.001, and striatum, *t*(4694)=3.38, *p*<.001.

When not considering age, IQ, head motion, or cross-validation accuracy, MVPA reward representation coupling with reward expectations in the striatum continued to significantly associate with CBCL internalizing symptoms, *t*(4694)=3.22, *p*=.001.

*Tests of specificity for internalizing symptoms*

CBCL internalizing and externalizing symptom domains are highly correlated, *r*=.69, *p*<.001. As such, univariate models, identical to those presented in the main results section, in which externalizing symptoms were included as predictors demonstrated that externalizing symptoms were associated with decreased coupling between reward representations in the striatum and reward expectation: *t*(4577)=-2.76, *p*=.006. However, in a subsequent model in which both internalizing and externalizing symptoms were included simultaneously, only internalizing symptoms, *t*(4577), *p*=.021, and not externalizing symptoms, *t*(4577), *p* =.25, were related to coupling between striatal reward representations and reward expectations.

**Supplemental Figure Legends**

Supplemental Figure 1. Histograms depicting continuous distributions of (log transformed) CBCL internalizing symptoms (left) and CTQ total severity scores (right).

Supplemental Figure 2. Depictions of relationships between decomposed CBCL internalized symptoms and softmax βs (top panel) and coupling between MVPA reward representations and expected reward of the chosen arm from the computational model (bottom panel).

Supplemental Figure 3. Comparison of main findings between sites. Sites did not differ in cross-validation accuracy (A), relationships between softmax βs and MVPA reward representations and expected reward of the chosen arm from the computational model (B), or CBCL internalizing symptoms and coupling between MVPA reward representations and expected reward of the chosen arm from the computational model (C).


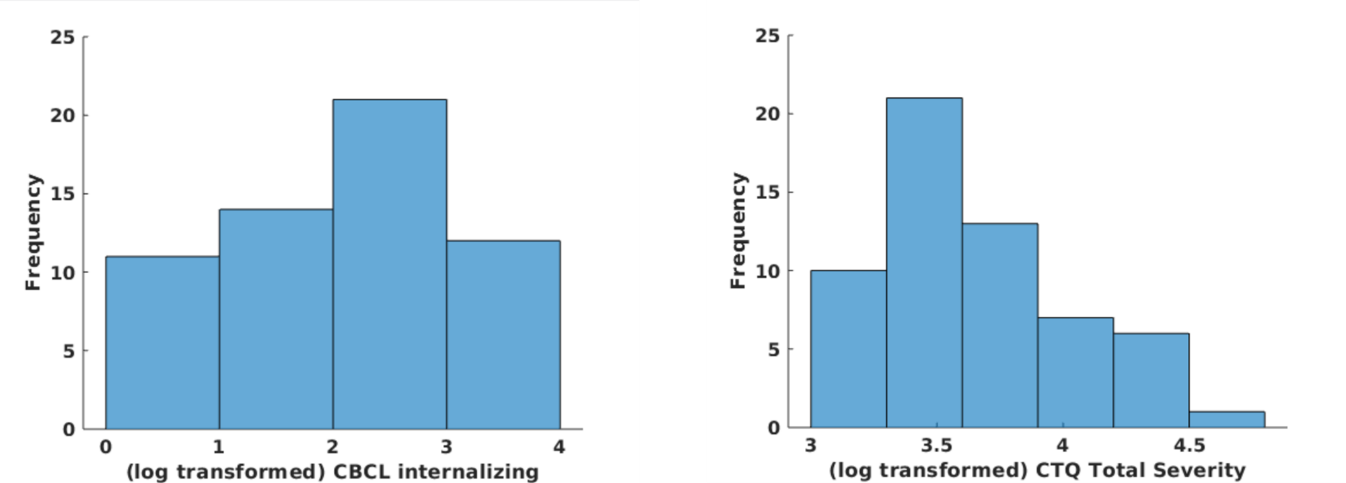


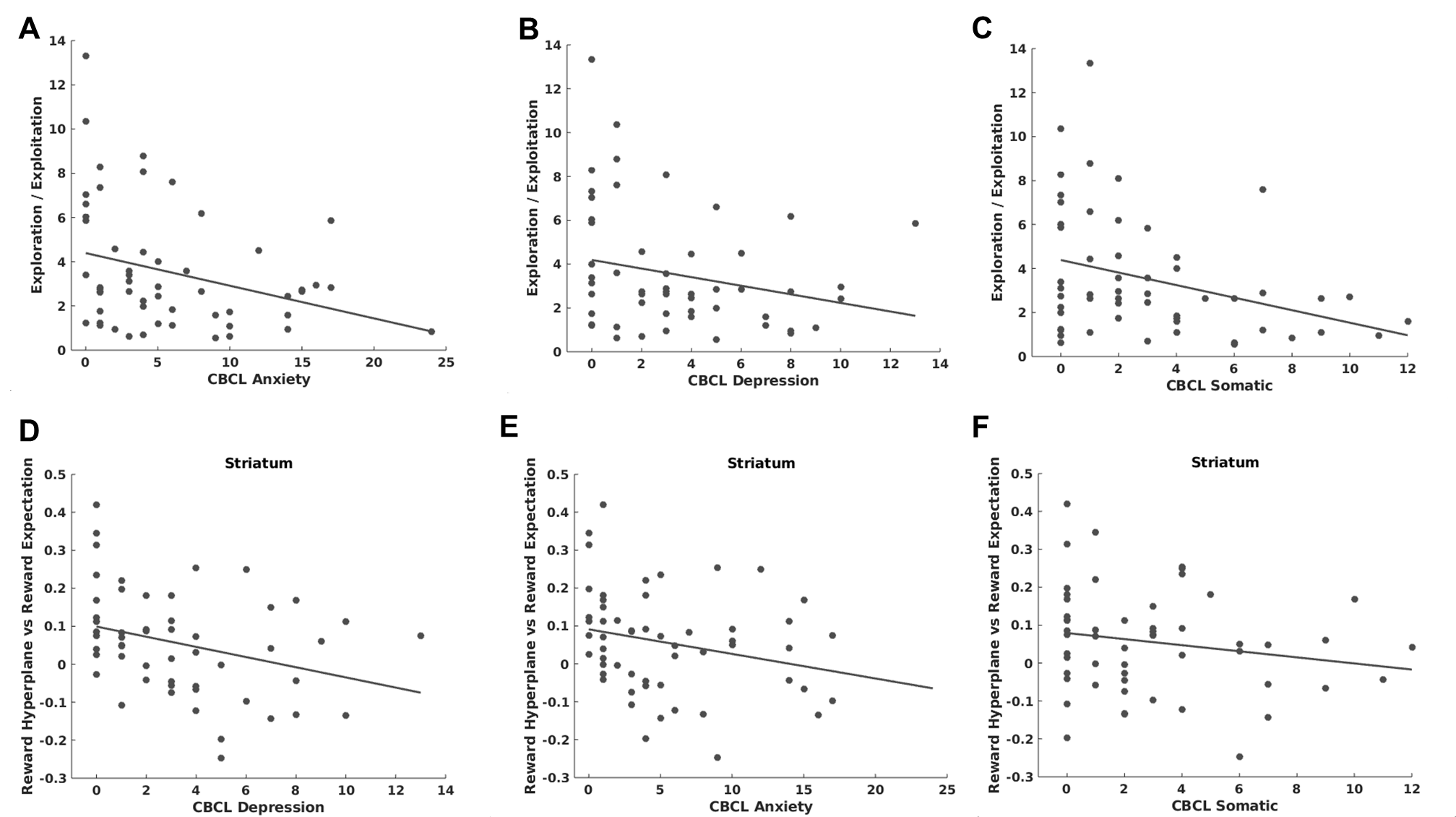


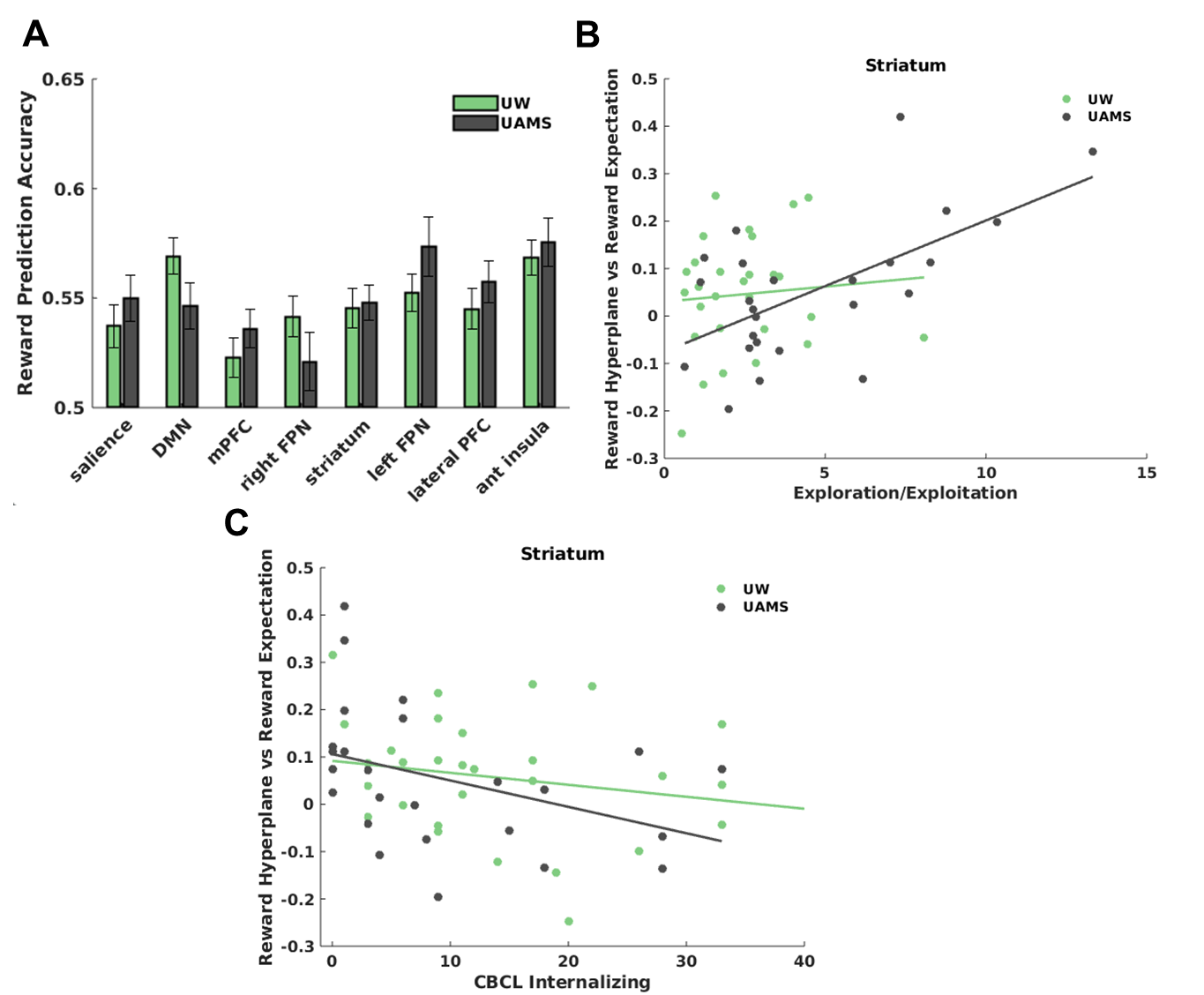


Piray, P., Dezfouli, A., Heskes, T., Frank, M. J., & Daw, N. D. (2019). Hierarchical Bayesian inference for concurrent model fitting and comparison for group studies. *PLOS Computational Biology*, *15*(6), e1007043. doi: 10.1371/journal.pcbi.1007043

Power, J. D., Mitra, A., Laumann, T. O., Snyder, A. Z., Schlaggar, B. L., & Petersen, S. E. (2014). Methods to detect, characterize, and remove motion artifact in resting state fMRI. *Neuroimage*, *84*, 320–341.

Siegel, J. S., Power, J. D., Dubis, J. W., Vogel, A. C., Church, J. A., Schlaggar, B. L., & Petersen, S. E. (2014). Statistical improvements in functional magnetic resonance imaging analyses produced by censoring high-motion data points. *Human Brain Mapping*, *35*(5), 1981–1996.
